# Docosahexaenoic Acid Induces Oxidative DNA Damage and Apoptosis, and Enhances the Chemosensitivity of Cancer Cells

**DOI:** 10.3390/ijms17081257

**Published:** 2016-08-03

**Authors:** Eun Ah Song, Hyeyoung Kim

**Affiliations:** Department of Food and Nutrition, Brain Korea 21 PLUS Project, College of Human Ecology, Yonsei University, Seoul 03722, Korea; 534ssong@yonsei.ac.kr

**Keywords:** docosahexaenoic acid, oxidative DNA damage, apoptosis, chemosensitivity, cancer cells

## Abstract

The human diet contains low amounts of ω-3 polyunsaturated fatty acids (PUFAs) and high amounts of ω-6 PUFAs, which has been reported to contribute to the incidence of cancer. Epidemiological studies have shown that a high consumption of fish oil or ω-3 PUFAs reduced the risk of colon, pancreatic, and endometrial cancers. The ω-3 PUFA, docosahexaenoic acid (DHA), shows anticancer activity by inducing apoptosis of some human cancer cells without toxicity against normal cells. DHA induces oxidative stress and oxidative DNA adduct formation by depleting intracellular glutathione (GSH) and decreasing the mitochondrial function of cancer cells. Oxidative DNA damage and DNA strand breaks activate DNA damage responses to repair the damaged DNA. However, excessive DNA damage beyond the capacity of the DNA repair processes may initiate apoptotic signaling pathways and cell cycle arrest in cancer cells. DHA shows a variable inhibitory effect on cancer cell growth depending on the cells’ molecular properties and degree of malignancy. It has been shown to affect DNA repair processes including DNA-dependent protein kinases and mismatch repair in cancer cells. Moreover, DHA enhanced the efficacy of anticancer drugs by increasing drug uptake and suppressing survival pathways in cancer cells. In this review, DHA-induced oxidative DNA damage, apoptotic signaling, and enhancement of chemosensitivity in cancer cells will be discussed based on recent studies.

## 1. Introduction

The human diet contains low amounts of ω-3 polyunsaturated fatty acids (PUFAs) and high amounts of ω-6 PUFAs, which might contribute to increased cancer incidence. In a previous study, breast cancer risk was positively associated with the ratio of dietary ω-6 to ω-3 PUFAs and inversely associated with the dietary intake of long-chain ω-3 PUFAs [1]. Studies conducted in humans have demonstrated that a high consumption of fish oil reduced the risk of cancer. In a Japanese cohort study, there was an inverse relationship between the risk of distal colon and pancreatic cancers and ω-3 PUFA consumption [2,3]. In a Scottish study, a significant reduction in colon cancer risk was associated with increased intake of total ω-3 PUFAs as well as eicosapentaenoic acid (EPA) or docosahexaenoic acid (DHA), consumed separately [4]. Chavarro et al. [5] analyzed the blood fatty acid levels of 476 men with prostate cancer during a 13-year follow-up and their matched controls. They found that whole blood levels of all long-chain ω-3 PUFAs were inversely related to overall prostate cancer risk. The blood levels of γ-linolenic and dihomo-γ-linolenic acids, fatty acids generated from the metabolism of linoleic acid, were directly associated with prostate cancer. In a human study evaluating the association between endometrial cancer risk and the intake of fatty acids and fish, the ratio of ω-6 to ω-3 PUFAs was inversely associated with the risk of endometrial cancer. Therefore, the dietary intake of EPA and DHA from foods and supplements may protect against the development of endometrial cancer [6]. Furthermore, regarding the relationship between the incidences of colorectal cancer (CRC) types such as proximal colon and distal colon cancer and ω-3 PUFA intake, high ω-3 PUFA was associated with a lower risk of proximal colon cancer and an unaltered or even increased risk of distal colon cancer [7,8]. Between 10% and 15% of CRCs display microsatellite instability (MSI) with predominance in the proximal colon [9,10,11], and the MSI was found to be induced by the loss of DNA mismatch repair (MMR) activity [12]. Song et al. [13] demonstrated that ω-3 PUFA intake inhibited inflammatory pathways associated with the development of tumors that arise from defective MMR. Therefore, ω-3 PUFA intake appears to be associated with a lower risk for MSI-high CRC but not microsatellite-stable (MSS) tumors. They suggested a potential role for ω-3 PUFAs in protecting against CRC through MMR.

Natural, synthetic, and biological agents have been developed to reduce or delay the occurrence of malignancy [14]. Certain agents trigger DNA damage followed by cancer cell death, which is critical for the maintenance of proper physiological processes including tissue homeostasis and immune function regulation [15,16,17]. However, dysregulation of cell death is often observed in cancer cells [18,19]. Therefore, the induction of cancer cell death is pivotal in cancer treatment, which makes it an important strategy in cancer therapy. 

The ω-3 PUFAs play vital roles in the normal growth and development of various cells and tissues [20,21]. DHA is one of the longest and most unsaturated fatty acids found in biological systems, with 22 carbons and six double bonds. DHA has been shown to inhibit cancer cell proliferation and induce death of some cancer cells [22,23,24,25]. DHA induces oxidative stress, DNA adduct formation, and DNA damage in various cancer cells [26,27], showing selective cytotoxicity against various types of cancer but not normal cells [28,29]. DHA favorably modulates anticancer treatment through its incorporation into cellular membranes, induction of oxidative stress, interaction with cellular signaling mediators, including cyclooxygenase-2, nuclear factor-κB, mitogen-activated protein kinases, and peroxisome proliferator-activated receptor-γ (PPARγ), and ability to increase the sensitivity of anticancer drugs in in vitro studies [30,31]. For patients with advanced non-small cell lung cancer undergoing platinum-based chemotherapy (carboplatin with vinorelbine or gemcitabine), supplementation with fish oil (2.5 g EPA + DHA/day) increased the chemotherapy efficacy without affecting the toxicity profile compared with the standard of care [32]. Bougnoux et al. [33] studied the effect of daily supplementation with 1.8 g DHA on the efficiency of an anthracycline-based chemotherapy regimen in patients with breast cancer. DHA during chemotherapy was devoid of adverse side effects and improved the outcome of chemotherapy when it was highly incorporated in the body. They suggested that DHA has the potential to specifically chemosensitize tumors. A pilot phase II clinical trial investigating the treatment of patients with metastatic breast cancer with dietary DHA (1.8 g/day) and an anthracycline-based chemotherapy revealed an improved survival rate, especially in a patient subpopulation with a high incorporation of DHA in the plasma. DHA had a specific chemosensitizing effect on metastases that was not observed in non-tumor tissues [34]. 

Although DHA shows chemopreventive activity as mentioned above, its potential anticancer effects are suggested in this review based on its oxidative stress–induced DNA damage, apoptosis, and the enhancement of chemosensitivity in cancer cells.

## 2. DHA Induces Oxidative Stress and Oxidative DNA Damage to Cancer Cells

High levels of reactive oxygen species (ROS) such as superoxide anions and hydroxyl radicals may induce oxidative stress in cancer cells. Superoxide anions react with nitric oxide (NO) to form peroxynitrite, a reactive nitrogen species (RNS). Both ROS and RNS induce DNA damage and DNA strand breaks. Merendino et al. [35] showed that DHA induced oxidative stress by depleting intracellular glutathione (GSH) in Paca-44 pancreatic cancer cells. They suggested that GSH depletion occurred via active GSH extrusion in cancer cells, since the inhibition of GSH efflux by treatment with the specific inhibitors of carrier-mediated GSH extrusion, cystathionine or methionine, completely reversed the apoptosis. Both EPA and DHA induced ROS accumulation and caspase-8–dependent apoptosis in breast cancer MCF-7 [36] and pancreatic cancer (MIA-PaCa-2 and Capan-2) cells in vitro. The growth of MIA-PaCa-2 human pancreatic cancer xenografts in athymic nude mice was suppressed by 5% fish oil supplementation, which induced oxidative stress and cell death [37]. Shin et al. [38] showed that DHA increased the cellular ROS levels and apoptosis of PC3 and DU145 prostate cancer cells expressing mutant p53. Pretreatment with the antioxidant *N*-acetyl-cysteine completely blocked the DHA-induced reduction in cell viability and reduced the elevated poly(ADP-ribose) polymerase cleavage caused by DHA. These findings suggest that DHA induces apoptosis by triggering intracellular ROS accumulation in these cells.

Mitochondria are a major source of intracellular ROS in mammalian cells. DHA has been shown to induce intracellular ROS by promoting the generation of mitochondrial ROS in certain cancer cells [39,40,41]. DHA induced excessive mitochondrial ROS accumulation in PA-1 human ovarian cancer cells [39] and human papillomavirus (HPV)-infected HeLa and SiHa human cervical cancer cells [40,41]. In both studies, the DHA-induced mitochondrial ROS overproduction was accompanied by mitochondrial malfunction, evidenced by the loss of mitochondrial membrane potential following the addition of DHA. Since the oxygen consumption rate was decreased by DHA treatment, DHA might trigger excessive mitochondrial ROS generation by disrupting the mitochondrial electron transport chain from producing ROS in cancer cells. 

DHA is oxidized by ROS, resulting in the generation of electrophilic compounds that have the potential to form DNA adducts, thereby initiating apoptotic responses in cancer cells [42]. DHA increased the formation of acrolein-derived 1,*N*^2^-propanodeoxyguanosine (Acr-dG), a major DNA adduct formed from oxidized DHA, and induced apoptosis. Interestingly, it was only after the Acr-dG reached a certain threshold level, which was beyond the capacity of nucleotide excision repair (NER) and other DNA repair pathways, that the cells underwent cell cycle arrest and induced apoptotic signaling pathways. In colon cancer HT-29 cells, DHA caused DNA adduct formation and cell cycle arrest in the G1 phase [43]. Since cancer cells produce relatively large amounts of ROS, DHA can be easily oxidized to form DNA adducts and cause DNA damage, which may result in the apoptosis of cancer cells. 

## 3. DHA Induces Apoptotic Signaling and Affects DNA Damage Response in Cancer Cells

When DNA damage occurs from diverse factors including oxidative stress and exogenous sources, cellular processes lead to cell cycle arrest and DNA damage repair as a defense mechanism [44]. Excess DNA damage, beyond the capacity of DNA repair, results in apoptotic cell death. Defects in the apoptotic pathway are widely observed in cancer cells. The pro-apoptotic effect of DHA on cancer cells has been widely documented both in vitro and in vivo in various types of cancer cells such as colon HT-29 [45], gastric AGS [46], pancreatic Paca-44 [47], lung 549 [48], and colorectal stem-like [49] cancer cells. 

DHA induced apoptosis in the human colon adenocarcinoma cell line HCT116, which carries an activating mutation of the β-catenin gene (*CTNNB1*), and SW480 cells with wild-type *CTNNB1* [50]. The proposed mechanisms of DHA did not involve the modification of the transcription of β-catenin, but induced ubiquitin-dependent proteasomal degradation of the protein was suggested [50]. In breast cancer lines MCF-7 and Hs578T, DHA pretreatment attenuated 12-*O*-tetradecanoylphorbol-13-acetate (TPA)-induced cell migration as well as protein kinase C δ (PKCδ), Wnt-1, and β-catenin expression. A study suggested that the anti-metastatic potential of DHA is partly attributable to its suppression of TPA-activated PKCδ and Wnt-1 signaling [51]. Xue et al. [52] conducted an investigation in BABL/c mice bearing breast cancer tumors. A 5% fish oil–supplemented diet for a period of 30 days significantly reduced the growth of 4T1 mouse breast cancer cells by downregulating β-catenin in tumor tissues with a notable increase in apoptosis. Fluckiger et al. [53] showed that DHA triggered apoptosis in the HCT-116 and HCT-8 human colorectal cancer cell lines in an autocrine tumor necrosis factor (TNF)-α–dependent manner. They demonstrated that DHA stimulated nuclear accumulation of Forkhead box O3 which binds to the microRNA-21 promoter. Therefore, DHA induced the mRNA expression of TNF-α through post-transcriptional regulation by the downregulation of microRNA-21 expression. A recent study compared DHA-induced stress responses in two human colon cancer cell lines, SW620 and Caco-2 [54]. DHA inhibited the growth of SW620 cells at early time points while that of the Caco-2 cells was unaffected by the same treatment. Furthermore, oxidative stress was induced in both cell lines, although at different time points and to varying extents. Therefore, the anticancer activity of DHA may differ depending on the molecular properties of the cancer cells.

Since DHA induces oxidative DNA damage in cancer cells, the DNA damage response may be affected by DHA in cancer cells. The p53 protein is a sensor at the center of the DNA damage response and is activated in response to multiple types of DNA damage. DHA was effective in the growth suppression of ovarian TOV-21G and breast MCF-10A cancer cells, which may be partly mediated by p53 activation [55,56]. Wan et al. [55] reported that EPA/DHA induced PPARγ and p53 overexpression in TOV-21G cells and the induction of p53 by EPA/DHA was abolished by the PPARγ antagonist GW9662. They found that the effect of DHA was more potent than that of EPA. The growth suppression of TOV-21G cells may be partly mediated by PPARγ and p53 activation during DHA treatment. Rescigno et al. [56] demonstrated that DHA differentially regulated the activation of extracellular signal-regulated kinase 1/2 (ERK1/2) and signal transducers and activators of transcription 3 (STAT3) pathways as well as cell cycle regulators such as p21 and p53 in breast cancer cell lines. DHA selectively arrested non-tumoral MCF-10A breast cells in the G0/G1 cell cycle phase by activation of p21 and p53. DHA induced cell death in highly transformed SK-BR-3 breast cells with the reduction of ERK1/2 and STAT3 phosphorylation, but only slightly affected the cell cycle in MCF-7 breast cancer cells with a transformation degree lower than that in the SK-BR-3 cells. These studies suggest DHA has a variable inhibitory effect on cancer cell growth that depends on the molecular properties and the degree of malignancy in each clinical case.

Kato et al. [57] compared the effect of DHA on the growth of the human colon carcinoma COLO 205 cells carrying wild-type p53 and WiDr colon carcinoma cells containing mutated p53 (His237). DHA inhibited the growth of COLO 205 cells by 81% and WiDr cells by 42%. DHA inhibited the proliferation of WiDr cells by 41%, but not that of COLO 205 cells. DHA arrested the cell cycle at the G0/G1 and G2/M phases in WiDr and COLO 205 cells, respectively. DHA induced the apoptosis of COLO 205 but not WiDr cells. Although DHA showed differential effects on cell proliferation, cell cycle arrest phase, and apoptosis in colon cancer cells depending on p53 status, it is evident that DHA inhibits cancer growth by p53-dependent and -independent pathways [57].

Experimental studies have demonstrated that inflammation inactivates MMR function and increases mutation rates [58,59,60]. The ω-6 PUFA-derived pro-inflammatory product prostaglandin E_2_ has been shown to silence DNA repair genes by enhancing DNA methylation to promote colonic tumor growth [61]. Since there is a strong inverse association of ω-3 PUFA with proximal colon cancer associated with defective MMR, the protective effect of ω-3 PUFAs against CRC is suggested to be mediated through DNA mismatch repair. On the other hand, oxidative stress inactivates MMR gene expression by mutation [59,62] or epigenetic silencing and may directly damage MMR proteins [63]. Since a high amount of DHA produces oxidative stress in cancer cells, DHA may inactivate MMR in cancer cells by ROS-dependent pathways.

## 4. DHA Increases Chemosensitivity of Cancer Cells

DHA has been found to enhance the activity of several anticancer drugs through an oxidative mechanism. Viet et al. [64] demonstrated that DHA increased the sensitivity of the breast cancer cell line MDA-MB-231 to doxorubicin, but it did not affect MCF-7 cells. In the MDA-MB-231 cells, DHA decreased the activity of cytosolic GSH peroxidase, an enzyme that protects against hydrogen and lipid peroxides. This modification of the GSH peroxidase response in the DHA-supplemented rats was associated with increased tumor sensitivity to anthracyclines. Therefore, the loss of the GSH peroxidase response due to oxidative stress in transformed cells may account for the ability of peroxidizable agents such as DHA to enhance tumor sensitivity to ROS-generating anticancer drugs.

Wang et al. [65] demonstrated that combined treatment with DHA and the anticancer drug etoposide exhibited an additive effect on brain tumor cells. Compared to etoposide used alone, the combination of DHA and etoposide suppressed the expression of the DNA-dependent protein kinase catalytic subunit (DNA-PKcs), which is involved in non-homologous end joining (NHEJ) repair pathways [66]. Recently, Chauvin et al. [67] investigated whether DHA increased tumor sensitivity to docetaxel by downregulating the survival pathways. Taxanes induce drug resistance by increasing the activity of phosphoinositide 3-kinase (PI3K)/Akt and ERK signaling pathways, which promote survival and cell growth of human cancer cells. In docetaxel-treated MDA-MB-231 cells, ERK1/2 phosphorylation and protein kinase C (PKC) activity were increased compared to that in untreated cells. In DHA-supplemented cells, docetaxel was unable to increase PKC levels in the membrane and nuclear fractions and diminished ERK1/2 phosphorylation, resulting in increased docetaxel efficacy.

DHA treatment affected the expression of the target proteins of cancer therapy such as p21, CDC25 homolog, and cyclin-dependent kinase 1, which led to cell cycle arrest at both the G1 and G2 phases in chemotherapy-resistant colon cancer SW620 cells [68]. The activity of P-glycoprotein (Pgp) and multidrug resistance-related protein 1 (MRP1), two membrane transporters involved in the multidrug resistance of colon cancer, is increased by high amounts of cholesterol in the plasma membrane and detergent-resistant membranes (DRMs). Multidrug-resistant (MDR) tumors, which overexpressed Pgp and MRP1, had a dysregulated cholesterol metabolism due to the lower expression of ubiquitin E3 ligase [69]. The ω-3 PUFAs were incorporated in the DRMs of MDR cells and they reduced the Pgp and MRP1 content of the DRMs, resulting in decreased transporter activity and restoration of the antitumor effects of the chemotherapeutic drugs.

The ω-3 PUFA conjugates of anticancer drugs have been the focus of increasing attention due to their enhancement of curative effects, reduction of side effects, and tumor-targeting abilities in preclinical studies. The ω-3 PUFAs are readily incorporated into the lipid bilayer of tumor cells and, therefore, can be used as a useful carrier to increase the therapeutic efficacy of anticancer drugs. Wang et al. [34] reported that the DHA-paclitaxel has received phase III clinical trial approval. Further research is necessary to determine specific mechanisms by which ω-3 PUFAs increase chemotherapy efficacy and to determine the optimal cellular/membrane levels of ω-3 PUFAs that increase the chemosensitivity and therapeutic efficacy of anticancer drugs.

## 5. Conclusions

**Figure 1:**
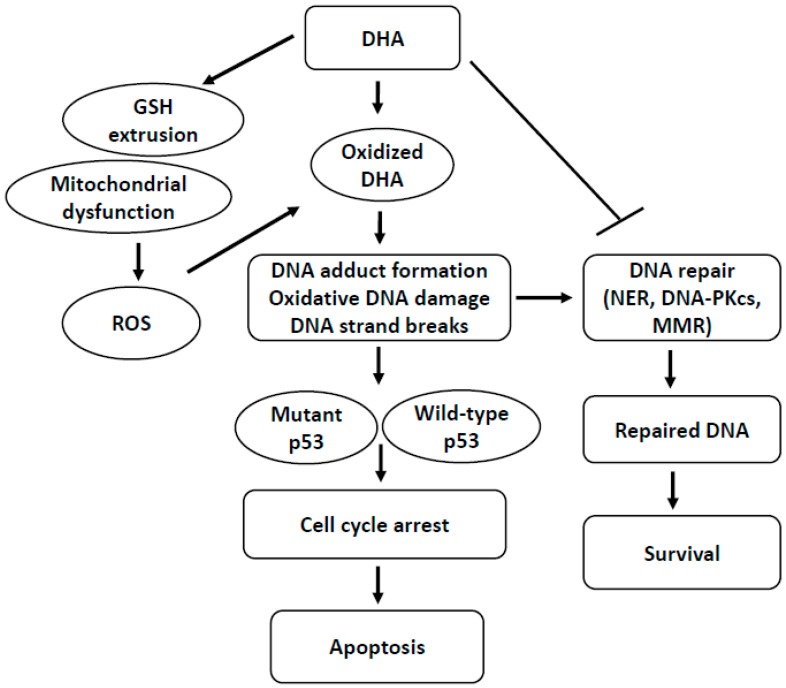
Proposed mechanism of docosahexaenoic acid (DHA)-induced DNA damage response in human cancer cells. DHA initially induces glutathione (GSH) extrusion and mitochondrial dysfunction, which increases reactive oxygen species (ROS) in cancer cells. DHA is oxidized, leading to DNA adduct formation and oxidative DNA damage which triggers cell cycle arrest and apoptosis in p53-dependent and p53-independent pathways. DNA damage is repaired by DNA repair processes such as nucleotide excision repair (NER), mismatch repair (MMR), and non-homologous end joining (NHEJ) mediated by DNA-dependent protein kinase catalytic subunit (DNA-PKcs). After the damaged DNA is repaired, the cells survive. However, excess DNA damage, induced by oxidized DHA, beyond the capacity of the DNA repair processes, initiates apoptotic signaling in cancer cells. Moreover, DHA suppresses the expression of DNA-PKcs and inactivates MMR in some cancer cells.

The induction of cancer cell death is important in cancer therapy. Recent studies have highlighted DHA as an effective anticancer agent because it induces apoptosis and enhances the drug sensitivity of cancer cells but not normal cells. In the cancer cell environment, DHA is oxidized to form DNA adducts such as Acr-dG and induces oxidative DNA damage, which triggers apoptosis of some cancer cells. DNA damage is repaired by DNA repair processes such as NER, MMR, and NHEJ including DNA-PKcs. However, excess DNA damage, induced by oxidized DHA, beyond the capacity of the DNA repair processes, initiates apoptotic signaling in cancer cells. Moreover, DHA suppresses the expression of DNA-PKcs in some cancer cells. DHA inactivates MMR in some cancer cells by ROS-dependent pathways (Figure 1). Therefore, DHA treatment or supplementation may be beneficial for cancer prevention and therapy. In addition, DHA increases tumor sensitivity to anticancer drugs by enhancing drug uptake and inhibiting survival signaling in cancer cells, as well as reducing Pgp and MRP1 in MDR tumors.
